# Reliability of Wireless Sensor Networks

**DOI:** 10.3390/s140915760

**Published:** 2014-08-25

**Authors:** Antônio Dâmaso, Nelson Rosa, Paulo Maciel

**Affiliations:** Centro de Informática, Universidade Federal de Pernambuco, Recife, PE, 50740-560, Brazil; E-Mails: avld@cin.ufpe.br (A.D.); prmm@cin.ufpe.br (P.M.)

**Keywords:** wireless sensor network, reliability, power consumption, reliability block diagram

## Abstract

Wireless Sensor Networks (WSNs) consist of hundreds or thousands of sensor nodes with limited processing, storage, and battery capabilities. There are several strategies to reduce the power consumption of WSN nodes (by increasing the network lifetime) and increase the reliability of the network (by improving the WSN Quality of Service). However, there is an inherent conflict between power consumption and reliability: an increase in reliability usually leads to an increase in power consumption. For example, routing algorithms can send the same packet though different paths (multipath strategy), which it is important for reliability, but they significantly increase the WSN power consumption. In this context, this paper proposes a model for evaluating the reliability of WSNs considering the battery level as a key factor. Moreover, this model is based on routing algorithms used by WSNs. In order to evaluate the proposed models, three scenarios were considered to show the impact of the power consumption on the reliability of WSNs.

## 1. Introduction

Wireless Sensor Networks (WSNs) consist of thousands of sensor nodes that collect data from the environment and send them to the sink node, which in turn forwards it to a remote user. WSNs have limited resources (e.g., battery, processing capacity), and should work as long as possible in the environment while collecting and sending data to a sink node.

Several strategies have been developed to optimize the power consumption (increasing the network lifetime) and increase the reliability (increasing the probability of a packet being delivered) of WSNs. However, strategies to decrease the power consumption negatively affect the reliability of the network (and *vice versa*). We can mention two strategies implemented in the protocol stack. In the first, part of the network should work while others should sleep. This strategy is excellent for power consumption, but not for reliability [1]. Part of the network may become inaccessible because a WSN node may be sleeping. The second strategy creates multiple paths (via a routing algorithm) between a particular WSN node and the sink node. Unlike the previous strategy, this strategy is excellent for reliability, but not for the power consumption; because it will use more than one way (more WSN nodes) to transmit the same packet. Hence, it is important to evaluate the WSN reliability considering the power consumption.

One way to evaluate the reliability of WSN is doing experiments with actual WSN nodes, inserting faults artificially [2] or analyzing them in actual scenarios [3]. For example, Zhao and Govindan [4] analyzed the impact of physical and MAC layers on the reliability of a WSN under different conditions (e.g., indoors and outdoors). However, these experiments tend to be boring, because they are manual and require a long time to get the data. The other way to evaluate the reliability is by using simulation or analytical modeling. Works on simulation evaluate the WSN reliability by expressing the network behavior (e.g., create, send, receive, forward and discard packets). They use models that consider irregular propagation and interference by other elements (e.g., Radio Irregularity Model [5]) and which packets are discarded mainly due to environmental conditions (e.g., noise, interference). This is ideal to define and evaluate communication protocols. For example, Wang *et al.* [6] proposed a congestion control protocol called Priority-based Congestion Control Protocol (PCCP) for WSNs. However, this work does not consider that a fault can occur in the sensor node, and a simulation can take a long time to evaluate the entire network. These disadvantages are solved by analytical modeling, which evaluates the network through mathematical representations, such as Markov Chain, RBD and Tree Fault [1]. For example, Bein *et al.* [7] evaluated the cost and availability of a WSN using Markov Chain and Ghaffari *et al.* [8] used RBD for evaluating the reliability of two transport protocols (ESRT and RMST).

However, these models consider fixed reliability values for all WSN nodes and evaluate the reliability of the entire WSN (instead of part of the network). The methodology proposed by Silva *et al.* [9] does not have some these drawbacks; however, they do not consider battery level as a factor that affects the sensor node reliability and they did not assess the power consumption before evaluating the reliability. Additionally, they do not build reliability models considering the routing algorithm used in the WSN in such way that is difficult to evaluate some existing strategies (like multiple paths). Additionally, this model does not consider that different routing protocols interfere with the reliability of WSN.

In this context, this paper proposes a model (called Region Model) to evaluate the reliability of each part (region) of a WSN. The proposed model is constructed considering routing algorithms used in WSNs. Hence, it is possible to observe which WSN region needs more attention and demands particular actions like the addition of more sensor nodes or changing the routing algorithm. Furthermore, the battery level is also explicitly considered as a key factor to evaluate the WSN reliability. A tooling is also proposed to facilitate the development and automate the evaluation of the WSN reliability.

This paper has three unique contributions. The first one is the proposed reliability model considering both the routing algorithms and the power consumption; the second contribution is the automatic generation of WSN reliability models; and the last one is the proposed tooling to support the reliability analysis.

This paper is structured as follows: Section 2 introduces basic concepts about WSNs, power consumption models and the Reliability Block Diagram (RBD); Section 3 shows how to create the Region Model to assess the reliability of WSNs; the next section, Section 4 presents three experiments to validate the proposed model; Section 5 presents related works that also assess the reliability of WSNs; and, finally, Section 6 presents the conclusions and some future works.

## 2. Background

In this section, we introduce some basic concepts in order to help to understand the rest of the paper. Firstly, it presents features, protocols and failures of WSNs. Next, it describes the power consumption models used in the reliability evaluation process of WSNs. Finally, it presents the formalism used to model the reliability, namely RBD.

### 2.1. Wireless Sensor Networks (WSNs)

Due to the advances in micro-electromechanical systems (MEMs), it has been possible to create sensor nodes that are small in size, have limited resources (sensing, data processing, transceiver and non-rechargeable batteries), and are able to communicate over short distances [10]. Hundreds or even thousands of sensor nodes usually make up a WSN. Due to their limited battery capacity, WSN nodes must consume little energy in order to keep the WSN lifetime as long as possible. The usual large number of WSN nodes favors the use of multi-hop communication (as opposed to using long-range communication) to save energy in data transmissions inside the WSN [11].

**Figure 1.**
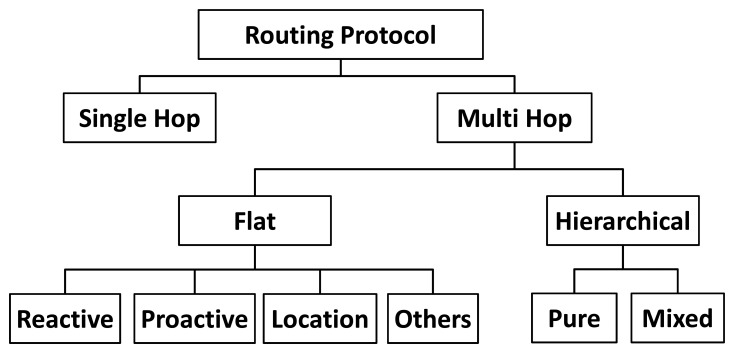
Taxonomy of routing protocols.

Routing protocols are responsible for transferring data packets from a source node to a target node through the network [12]. They use routing algorithms, which are responsible for determining the path (next nodes) that a packet should be forwarded along [13]. As illustrated in Figure 1, routing protocols can be single hop, *i.e.*, a packet is directly sent from the source to target, or multi hop, *i.e.*, a packet is sent to the target through several nodes. Multi hop protocols can be divided in flat, in which all nodes have the same responsibility, or hierarchical, that creates clusters inside the network. A flat routing protocol adopts different strategies to create a path. It can determine a path when: (i) a sensor node dies or is started (Reactive); (ii) when a packet should be sent (Proactive); (iii) when it receives the location of the others sensor nodes (Location); (iv) or any other strategy (Others). In hierarchical protocols, each cluster has a leader (Cluster Head—CH) and several participants. In the case the cluster is purely hierarchical, the participants of a cluster send packets to their CHs, which forwards the packet to the sink node. Otherwise (in a mixed hierarchical), the network is flat inside and/or outside the cluster: participants collaborate to deliver a packet to their CH that forwards it to another CH or to the sink node.

Whatever the strategy, packets do not arrive to the sink node if the path fails, which may occur in the communication link or in the WSN node. A link failure can happen due to noise, interference, distance (e.g., weak signal), or environmental conditions (e.g., walls). Meanwhile, a WSN node can fail due to software (e.g., application) or hardware (e.g., radio) failures.

Additionally, routing protocols can use a multipath strategy instead of a single path. In this case, the same packet is sent through different paths (normally called disjoint paths), which increases the probability of being delivered [14]. However, multipath mode uses more sensor nodes and, consequently, requires more power consumption (shorting the network lifetime) than single paths.

### 2.2. WSN Power Consumption Evaluation

**Figure 2.**
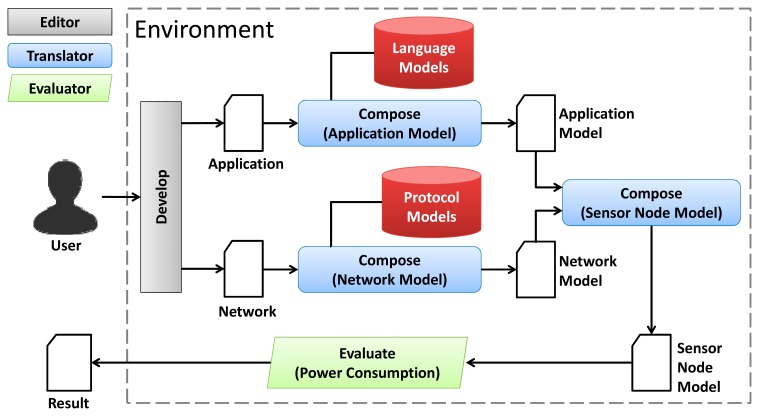
Step-by-step to evaluate the power consumption of WSNs.

The evaluation of the WSN power consumption is as important to assess as its reliability. WSN power consumption may be evaluated as proposed in [15,16]. In this approach, Coloured Petri Net (CPN) [17–19] models are used to evaluate the WSN power consumption through a set of steps as shown in Figure 2.

**Figure 3.**
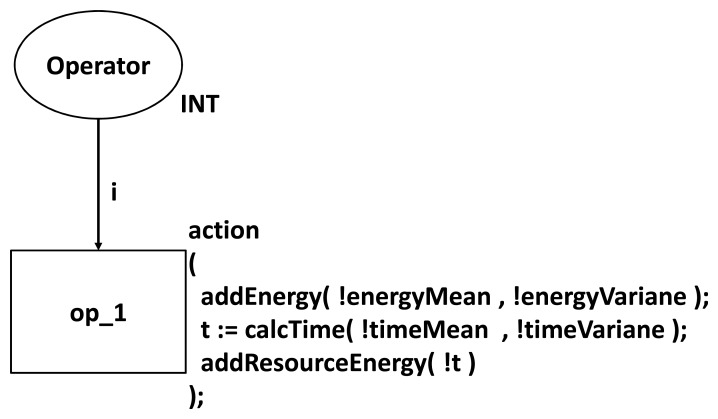
Power consumption model of the nesC assignment operator.

In the first step, the user implements an application (in a programming language) and configures the WSN by defining its topology and adopted communication protocols. Next, the application code and network configurations are converted into Application and Network CPN Models, respectively. These models are composed by basic models (Language Model and Protocol Model), which represent the power consumption of small parts of the application (e.g., power consumption of a piece of code) or the network (e.g., power consumption of a link layer protocol). The next step is to integrate them into the Sensor Node Model in order to simultaneously evaluate the power consumption of the application and network. Figure 3 presents the CPN model used to represent the power consumption of an assignment command in a programming language like nesC [20]. When transition *op_1* is triggered, this model calculates the power consumption of this command. Additionally, the Application Model is composed of several language models connected sequentially.

### 2.3. Reliability Block Diagram

Consider a system S composed by a set of components, *C* = {*c_i_*|1 ≤ *i* ≤ *n*}, where the state of the system S and its components could be either operational or failed. Let the discrete random variable *x_i_* indicates the state of component *i*, thus:

$$x_{i}=\{\begin{array}{ll} 0 & \text{if the component i has failed}\\ 1 & \text{if the component i is operational} \end{array}$$

The vector **x** = (*x*_1_, *x*_2_, …, *x_i_*, …, *x_n_*). Wherever need and the context is clear, **x** may also be referred to as a set that represents the state of each component of the system, and it is named state vector. The system state may be represented by a discrete random variable *ϕ*(**x**) = *ϕ*(*x*_1_, *x*_2_, …, *x_i_*, …*x_n_*), such that:

$$\phi (\text{x})=\{\begin{array}{ll} 0 & \text{if the system has failed}\\ 1 & \text{if the system is operational} \end{array}$$

*ϕ*(**x**) is called the structure function of the system. For any component *c_i_*:

(1) $$\phi (\text{x})=x_{i}\phi (1_{i},\text{x})+(1-x_{i})\phi (0_{i},\text{x}),$$

where *ϕ*(1*_i_*, **x**) = *ϕ*(*x*_1_, *x*_2_, …, 1*_i_*, …, *x_n_*) and *ϕ*(0*_i_*, **x**) = *ϕ*(*x*_1_, *x*_2_, …, 0*_i_*, …, *x_n_*).

Equation (1) expresses the system structure function in terms of two conditions. The first term (*x_i_ ϕ*(1*_i_*,**x**)) represents a state where the component *c_i_* is operational and the state of the other components are random variables (*ϕ*(*x*_1_, *x*_2_, …, 1*_i_*, …, *x_n_*)). The second term ((1 − *x_i_*) *ϕ*(0*_i_*, **x**)), on the other hand, states the condition where the component *c_i_* has failed and the state of the other components are random variables (*ϕ*(*x*_1_, *x*_2_, …, 0*_i_*, …, *x_n_*)). Equation (1) is known as factoring of the structure function and very useful for studying complex system structures, since through its repeated application one can eventually reach a subsystem whose structure function is simple to deal with Equation (1).

If one is interested in representing the system state at a specific time *t*, the components' state variables should be interpreted as a random variables at time *t*. Hence, *ϕ*(**x**(t)), where x(t) = (*x*_1_(*t*), *x*_2_(*t*), …, *x_i_*(*t*), …*x_n_*(*t*)).

The most straightforward strategy for computing system reliability of system composed of independent components is through its respective structure function *ϕ*(**x**(t)). The system reliability is defined by *R_S_*(*t*) ⩵ *P*{*ϕ*(**x**(t)) = 1}, where *P{c}* denotes the probability a given condition *c*. Since *ϕ*(**x**(t)) is a Bernoulli random variable, then *P*{*ϕ*(**x**(t)) = 1} = *E*[*ϕ*(**x**(t))], where *E[X]* is the expected value of the random variable *X*, therefore, *R_S_*(t) = *E*[*ϕ*(**x**(t))].

Reliability Block Diagram (RBD) is the most adopted notation to express and assess the reliability of systems [1,21,22]. RBDs enable us to represent and evaluate the reliability of a system by composing a set of blocks, where each block represents the reliability of an element of the system, e.g., the reliability of a router.

**Figure 4.**
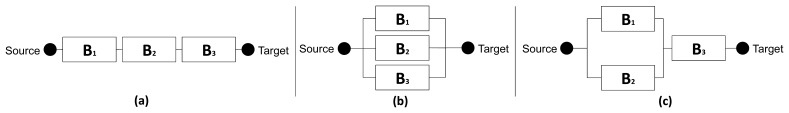
RBD organized in (**a**) series; (**b**) parallel and (**c**) combined.

As shown in Figure 4, the RBD has input and output points, which represent the beginning and the end of the system being evaluated, respectively. Blocks are arranged in series, parallel or combining them. These blocks have only two states (failure or working) and the failures are independent from each other. Each block has a reliability associated: block *B_i_* has its reliability *R_i_*(*t*) associated to “working state” at time *t*. Similarly, its complement (*1 − R_i_*(*t*)) represents the “failure state” at time *t*.

If for a system correctly functioning, every of its components should properly operate, the respective system RBD should be represented as a series its system components. Consider a **series structure** (as the one depicted in Figure 4a) composed of *n* **independent** components, where *p_i_*(*t*) = *P*{*x_i_*(*t*) = 1} are the functioning probabilities of blocks *b_i_*. These probabilities could be reliabilities or availabilities, for instance. The probability for the system to be operational is:

(2) $$P\{\phi (\text{x}(t))=1\}=P\{\phi (x_{1}(t),x_{2}(t),\ldots ,x_{i}(t),\ldots ,x_{n}(t))=1\}=\overset{n}{\underset{i=1}{\Pi }}P\{x_{i}(t)=1\}=\overset{n}{\underset{i=1}{\Pi }}p_{i}(t)=1$$

Therefore, the system reliability is:

(3) $$R_{S}(t)=P\{\phi (\text{x}(\text{t}))=1\}=\overset{n}{\underset{i=1}{\Pi }}P\{x_{i}(t)=1\}=\overset{n}{\underset{i=1}{\Pi }}R_{i}(t)$$

where *R_i_*(*t*) is the reliability of block *b_i_*.

Therefore, a system with *n* components in series (as shown in Figure 4a) has reliability (at time *t*) equal to the product of the reliability of the blocks that compose it, as shown in Equation (4):

(4) $$R(t)=\overset{n}{\underset{i=1}{\Pi }}R_{i}(t)$$

For example, an RBD with three blocks (each having a reliability of 0.9) has reliability equal to 0.729. On the other hand, if a system, composed by *n* components, works if at least one component properly works, the respective system RBD is arranged in parallel. Hence, a system with *n* components (as shown in Figure 4b) has reliability equal to the complement of products of unreliabilities of all blocks at time *t* (as shown in Equation (5)):

(5) $$R(t)=1-\overset{n}{\underset{i=1}{\Pi }}(1-R_{i}(t))$$

For example, the reliability of a system with three blocks (each having its reliability set to 0.9) is equal to 0.999. Additionally, it is important to note how the organization of the blocks influences the reliability of the whole system.

In addition to these two organizations, it is possible to combine blocks, creating other formats: series-parallel, parallel-series, bridge and *k-out-of-n*. These formats use Equations (4) and (5) to calculate the system reliability. For example, Figure 4c represents a RBD series-parallel where blocks *B1* and *B2* are in parallel, and *B3* is in series. First, it is necessary to solve the reliability of the parallel blocks and then the series one. Thus, if each block has reliability equal to 0.9, the reliability of the system is 0.891.

In some cases, simplifying the structure function may not be an easy task. Therefore, a logic function may be adopted to simplify system's functions through Boolean algebra. Using the Boolean notation, a Boolean variable *s_i_* is equivalent to *x_i_*, its complement $\bar{s_{i}}$ is represented by 1 − *x_i_*, the value *ϕ*(**x**) = 1 is represented by a “true” predicated *ϕ*(**bs**), and *ϕ*(**x**) = 0 value by the respective complement $\bar{\varphi (\text{bs})}$. Besides, ∧ represents ×, and ∨ is the respective counterpart of +.

#### 2.3.1. Minimal Paths and Minimal Cuts

Minimal path is a set of components organized in series whose guarantees system operation [23]. On the other hand, minimal cut is a set of components whose implies system failure [1,22,23].

For example, Figure 4a has only one minimal path ({B_1_B_2_B_3_}) and has three minimal cuts ({B_1_},{B_2_},{B_3_}).

#### 2.3.2. Sum of Disjoint Products (SDP)

The Sum of Disjoint Products (SDP) method is based on Boolean algebra and evaluates the probability of system operation by the union of the minimal paths or system failure by the union of the minimal cuts. We will call minimal path and minimal cut as event to facilitate explanation of the example. If two or more events have no components in common, the probability of at least one of the events will occur is the sum of the probabilities of the individual events [22,23]. If two events A and B have components in common, we have the following equation for evaluation of the probability of union of the events A and B (A ∪ B) [23]:

(6) $$R(A\cup B)=R(A)+R(\bar{A}B)$$

In this way, a system with *n* events (*A_1_*, *A_2_*, … , *A_n_*) has:

(7) $$R(A_{1}\cup A_{2}\cup \ldots \cup A_{3})=R(A_{1})+R(\bar{A_{1}}A_{2})+\cdots +R(\bar{A_{1}}\bar{A_{2}}\cdots \bar{A_{\left(n-1\right)}}A_{n})$$

## 3. Reliability Models of WSNs

This section presents the proposed approach to evaluate the reliability of WSNs considering their routing algorithms and power consumption. Initially, we introduce essential elements used in the definition of the reliability models. Next, we present the blocks and models used to express the reliability of these essential elements. By using these models, we present the impact of routing algorithms on the reliability of WSNs. Finally, we describe the tooling implemented to support these ideas.

### 3.1. Basic Definitions

**Figure 5.**
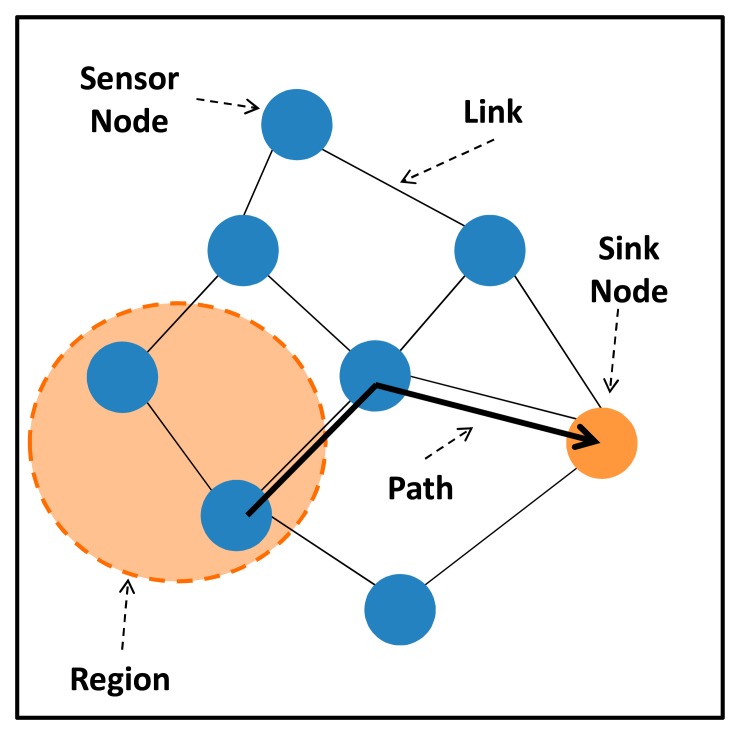
WSN Elements considered in the modeling process.

Prior to present the reliability models of a WSN, it is necessary to characterize the elements that we are considering in the modeling process. Figure 5 shows these elements and their relationships. Every WSN is composed by simple sensor nodes, sink nodes and communication links as introduced in Section 2.1. In addition to these elements, we adopt the notion of “WSN region”, which consists of a set of WSN nodes that sense the same physical phenomenon, and “WSN path” that represents a logical way (a set of WSN nodes and links) from a WSN node belonging to a particular region and the sink node. At this point, it is worth observing a key aspect in our approach: the WSN path depends on the routing algorithm being used. Hence, several WSN paths may exist between a particular node and the sink node and their reliability may be different.

**Figure 6.**
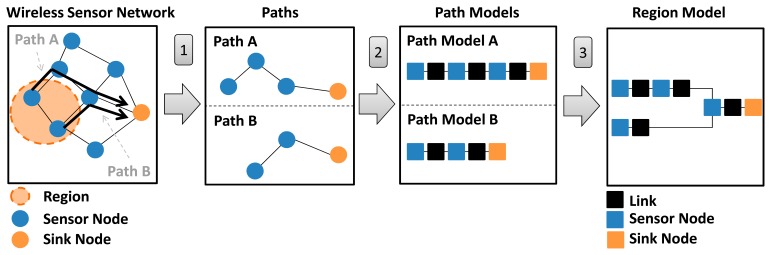
Overview of creating the Region Model.

By using these elements, we defined a strategy for modeling them in RBD. Firstly, each RBD model has a source (starting point) and a target (ending point); in WSNs, the starting point is any region in the network, whilst the ending point is always the sink node. Secondly, for reliability purpose, the WSN is divided into regions and its reliability is computed individually for each region. This division is justified because the reliability of a region is affected by four factors: positions of the WSN nodes that make up the region, routing algorithm used, reliability of the WSN nodes, and reliability of WSN links. Finally, each aforementioned element has associated a reliability model, which leads to the definition of Basic reliability blocks (used to model WSN nodes and links), Region model (to express the reliability of a WSN region) and a Path model (to specify the reliability of a WSN path). Figure 6 presents a step-by-step diagram of the proposed strategy.

Starting from a WSN configuration (topology and adopted routing algorithm), it is necessary to manually define a WSN region to be evaluated. Next steps are performed automatically (by the developed tooling) and consist of create WSN paths for each sensor nodes belonging to the region being evaluated. With the paths already defined, next step generates reliability models for each of them. Last step consists of composing the path models to produce the reliability model of the WSN region. Again, it is worth observing that the nodes that compose a path depend on the routing algorithm being utilized in the WSN. The following subsections present details of Basic block, Path Models and Region Models.

### 3.2. Basic Blocks

**Figure 7.**
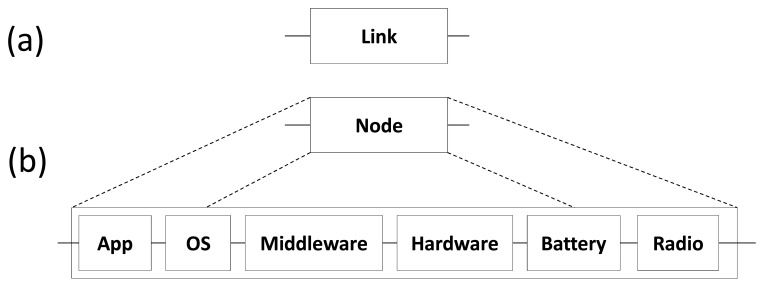
Basic blocks of the reliability model.

The reliability of communication links (block *Link*) and WSN nodes (block *Node*) are modeled by using RBD blocks as shown in Figure 7.

The reliability of a node is defined as a sequence of the following blocks: application (*App*), operating system (*OS*), middleware (*Middleware*), platform (*Hardware*), radio (*Radio*) and battery level (*Battery*). As the reliability of a WSN node is composed by the reliability in series of their components, if one of them fails, the whole node fails (see Section 2.3). Each block has associated a reliability defined by the user, obtained through simulation, or defined in the software/hardware specification.

In fact, the simulation is used to know the status (e.g., battery level, radio active/inactive) of a component at time *t*. By using this information (status), we define the component's reliability. For example, the node reliability is directly affected by its battery level, *i.e.*, low battery level means low reliability. Over time, the battery is consumed and can reach a level that is unable to meet the energy requirement of the node, which will not work properly (higher probability of fail) or will die (always fail). To estimate the battery reliability, its status is simulated to yields its reliability as defined in Section 4.1.

Similarly, the reliability of the radio is also defined by simulation. The radio status assumes two values: on and off. The node can send, receive or forward a packet when the radio is turned on. However, when it is turned off, the node becomes “inactive” and cannot send, receive or forward packets. A packet is lost if it is forwarded to a sensor node with radio turned off. To represent this situation in RBD, the block *Radio* should be used to indicate whether radio is on (reliability > 0.0) or off (reliability = 0.0) and its status at time *t* can be captured via simulation.

### 3.3. Path Model

The path model is defined by composing basic blocks, *i.e.*, node and link reliability blocks. A path includes at least two nodes (one hop), where one node starts (source) and another terminates (target) the path model. If there are more nodes involved (multiple-hop), they will be placed between the source and target blocks.

**Figure 8.**
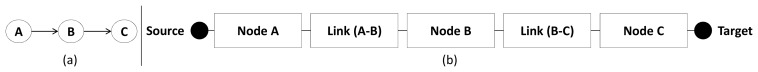
Example of a (**a**) WSN path and (**b**) its RBD model.

Figure 8a illustrates a path with sensor nodes *A*, *B* and *C* where the sensor node *A* is the source, the sensor node *C* is the target, and the sensor node *B* routes the packet between the source and target. The corresponding RBD model is illustrated in Figure 8b.

As RBD is a success oriented model, all system components, that is, *Node A, Link* (*A−B*), *Node B, Link* (*B−C*), and *Node C* should be arranged in series, since if at least one component fails the whole system fails. Additionally, this RBD model has only one minimal path (*MP* = {*Node A*, *Link* (*A* − *B*), *Node B*, *Link* (*B* − *C*), *Node C*}) .

**Figure 9.**
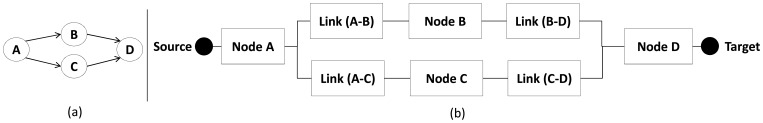
Example of a (**a**) network with multipath and (**b**) its RBD model.

A sensor node can also send or forward a packet through multiple paths as shown in Figure 9a. In this case, node *A* sends packets in broadcast, which are sent to nodes *B* and *C*. Nodes *B* and *C* send the packet to the same target (node *D*).

The RBD model of the network illustrated in Figure 9a has two or more paths in parallel where two or more nodes forward the same packet from the same transmitter. For building the RBD model, lets first name the basic blocks: *Node A, Node B, Node C, Node D, Link* (*A* − *B*), *Link* (*A* − *C*), *Link* (*C* − *D*) and *Link* (*B* − *D*). The RBD is depicted by two minimal paths (a minimal path is a path that is not composed of any other path): *MP*_1_ = {*Node A*, *Link* (*A* − *B*), *Node B*, *Link* (*B* − *D*), *Node D*} and *MP*_2_ = {*Node A*, *Link* (*A* − *C*), *Node C*, *Link* (*C* − *D*), *Node D*} . This RBD is shown in Figure 9b. It is worth observing that the path model depends on the routing algorithm used (see Section 3.5), because it determines which nodes will participate (and how to participate) in the path.

### 3.4. Region Model

**Figure 10.**
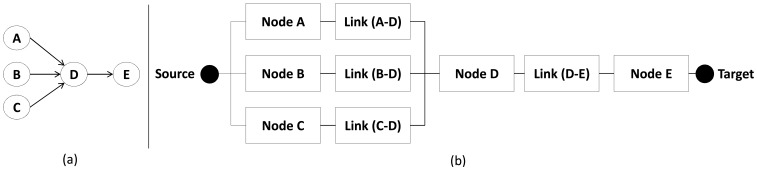
Example of a (**a**) region and its (**b**) region model.

As defined in Section 3.1, a region has one or more sensor nodes. In the case the region has just one node, the region and path models are the same. Otherwise, if the region has more than one sensor node, it is necessary to combine all path models of the sensor nodes belonging to the region. Before combining them, it is necessary to assess whether these paths shared nodes or not. For example, the region in Figure 10a has three nodes, namely *A*, *B* and *C*; consequently, it has three path models (A→D→E, B→D→E and C→D→E), which share two nodes (*D* and *E*). Figure 10b shows the corresponding reliability model to this region when we combine the path models. Additionally, the sensor nodes inside of region (*A*, *B* and *C*) are considered as the sources and sensor node *E* is considered as the target.

Assuming that the WSN shown in Figure 10a uses a hierarchical algorithm (see Section 2.1) and the sensor node *D* is the Cluster Head (CH) of sensor nodes *A*, *B* and *C*, all nodes in that region (*A*, *B* and *C*) should send their packets to the node *D* (CH). This means that all sensor nodes have one thing in common (node *D*) and this fact should be represented in the reliability model. If this common point fails, the region will fail independently from the existing paths. Therefore, lets first label the basic blocks: *Node A, Node B, Node C, Node D, Node E, Link* (*A* − *D*), *Link* (*B* − *D*), *Link* (*C* − *D*) and *Link* (*DE*). The RBD is represented by three minimal paths: *MP*_1_ = {*Node A*, *Link* (*A* − *D*), *Node D*, *Link* (*D* − *E*), *Node E*}, *MP*_2_ = {*Node B*, *Link* (*B* − *D*), *Node D*, *Link* (*D* − *E*), *Node E*} and *MP*_3_ = {*Node C*, *Link* (*C* − *D*), *Node D*, *Link* (*D* − *E*), *Node E*}. This RBD is depicted in Figure 10b.

**Figure 11.**
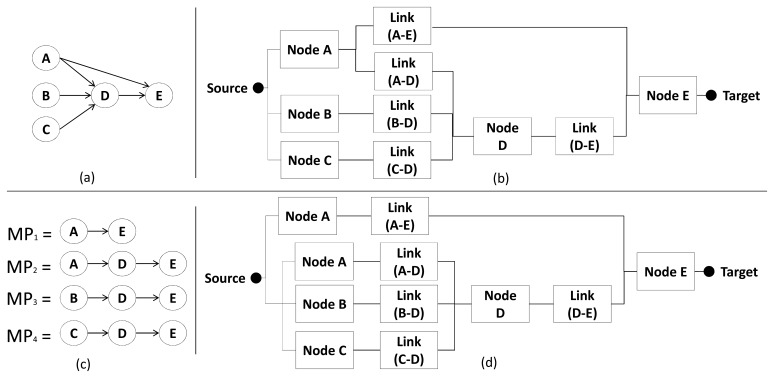
Specific scenario that depicts an (**a**) region; (**b**) no-series-parallel RBD; (**c**) minimal paths of the region; and (**d**) series-parallel RBD equivalent representation obtained as the SPD resulting logical function.

In some specific scenarios, as shown in Figure 11a, the operational behavior may not be represented by a series-parallel RBD composition. In such cases “pivoting” or SDP [22,23] methods should be applied compute the measures of interest.

For instance, the node *A* sends packets in multicast to nodes *D* and *E*, creating a no-series-parallel RBD composition—as shown in Figure 11b. To solve this situation, we need use only the SDP method, but it is necessary first to define minimal paths (see Section 2.3.1) as shown in Figure 11c. As these minimal paths have elements in common (*Node A*, *Node D* and *Node E*), the region reliability using SDP method is calculated as follows (see Section 2.3.2):

(8) $$\begin{array}{l} R_{\text{Region}}=R_{MP_{1}}+\bar{R_{MP_{1}}}R_{MP_{2}}+\bar{R_{MP_{1}}}\bar{R_{MP_{2}}}R_{MP_{3}}+\bar{R_{MP_{1}}}\bar{R_{MP_{2}}}\bar{R_{MP_{3}}}R_{MP_{4}}\\ =R_{A}R_{L(AE)}R_{E}\\ +\bar{R_{A}R_{L(AE)}R_{E}}R_{A}R_{L(AD)}R_{D}R_{L(DE)}R_{E}\\ +\bar{R_{A}R_{L(AE)}R_{E}}\bar{R_{A}R_{L\left(AD\right)}R_{D}R_{L\left(DE\right)}R_{E}}R_{B}R_{L(BD)}R_{D}R_{L(DE)}R_{E}\\ +\bar{R_{A}R_{L(AE)}R_{E}}\bar{R_{A}R_{L(AD)}R_{D}R_{L(DE)}R_{E}}\bar{R_{B}R_{L(BD)}R_{D}R_{L(DE)}R_{E}}R_{C}R_{L(CE)}R_{D}R_{L(DE)}R\\ =R_{E}(R_{A}R_{L(AE)}+R_{D}R_{L(DE)}(R_{C}R_{L(CD)}+R_{B}R_{L(BD)}+R_{A}R_{L(AD)})) \end{array}$$

where *MP* represents a minimal path illustrated in Figure 11c and *R_x_* represents the reliability of a node *x* (*i.e.*, *R_A_* is reliability of node *A*), link (*i.e.*, *R_L(AE)_* is reliability of link between node *A* and *E*), minimal path (*i.e.*, *R_MP1_* is reliability of minimal path *MP_1_*) or region (*R_Region_*). Applying the SDP method (see Section 2.3.2), we obtained the expression *R_E_* (*R_A_ R_L_*_(_*_AE_*_)_ + *R_D_ R_L_*_(_*_DE_*_)_ (*R_C_ R_L_*_(_*_CD_*_)_ + *R_B_ R_L_*_(_*_BD_*_)_ + *R_A_ R_L_*_(_*_AD_*_)_))). Additionally, we can create a series-parallel RBD model representing the reliability of this region—as illustrated in Figure 11d—based on the SDP expression. However, this additional step is not necessary to evaluate this region, because the method SDP is sufficient.

### 3.5. Impact of Routing on the WSN Reliability

**Figure 12.**
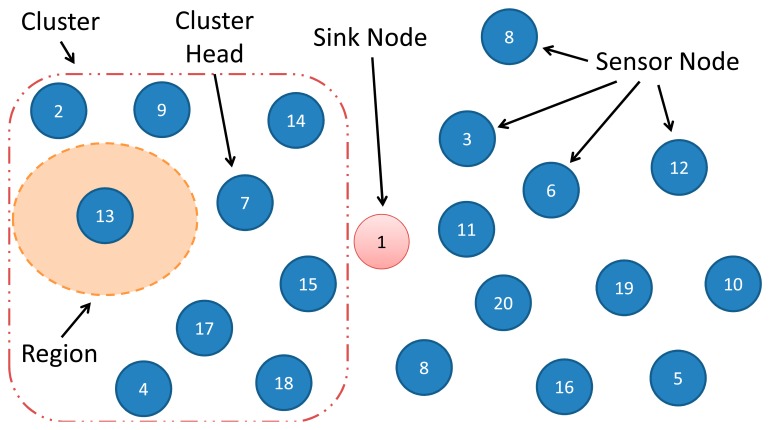
WSN region elements.

As mentioned in Section 3.1, the routing strategy has a key impact on the WSN reliability. Each routing algorithm defines a different set of nodes that compose the path from a region to the sink node. Hence, the reliability of a particular region depends on the adopted routing algorithm. Figure 12 illustrates a WSN used to describe the Region Models created by different routing algorithms.

This WSN has 20 nodes, a sink node (node 1), and a region with only one sensor node (node 13). The reliability model of this region will be shown in the following considering different routing protocols: DIRECT (single hop), FLOODING (flat) and LEACH (hierarchical). These protocols were selected for the following reasons: each one represents a category of routing protocol (see Section 2.1); they are easy to implement; they are widely adopted; and they have different behaviors (they have different strategies to define a path). For example, LEACH works by creating clusters, while FLOODING creates multipath. Furthermore, these protocols were used to illustrate that the proposed reliability model is independent from the adopted routing protocol.

#### 3.5.1. DIRECT Protocol

**Figure 13.**
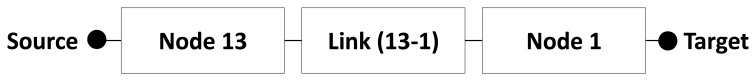
Region Model when the WSN uses the DIRECT protocol.

The DIRECT Protocol [24] is ideal for small networks where the sensor nodes can communicate directly with the sink node. Its routing algorithm selects only two sensors nodes: sender and sink node. Figure 13 illustrates the Region Model shown in Figure 11, when the WSN uses the DIRECT protocol. This model has only three blocks: *Node 13* (node belonging to the region); *Node 1* (sink node); and *Link (13-1)* modeling the link between them.

#### 3.5.2. FLOODING Protocol

**Figure 14.**
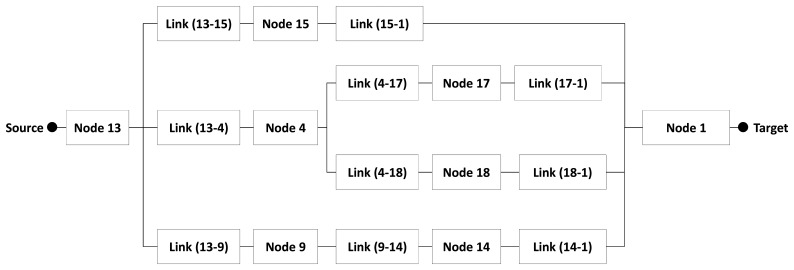
Region Model when the WSN uses the FLOODING protocol.

FLOODING sends packets in broadcast until reaching the sink node. We used a variant of this protocol (FLOODING with probability [25]) as it is less power consuming. Initially, a sensor node creates and sends a packet to its neighbors. When a neighbor receives this packet, it decides whether or not to forward the packet. To take this decision, it generates a random number and only forwards the packet if the number generated is less than a threshold *t* (which is defined by the user). Hence, the WSN will not use all sensor nodes of the network to forward a packet. Additionally, its routing algorithm uses the multipath strategy to send and forward a packet (Figure 14).

As mentioned before, the WSN has 20 nodes, but only 8 participated in the disjoint paths created in this case: block *Node 13* represents the region, block *Node 15*, *Node 4* and *Node 9* represent the sensor nodes (*Node 15*, *Node 4* and *Node 9*, respectively) that forward the same packet from *Node 13* and creating a multipath. The same occurs with blocks *Node 17* and *Node 18*, which represent the sensor nodes that send packets from node 4. This happened because all sensor nodes send the packet in broadcast. All these branches finish in block *Node 1* (sink node). Additionally, the region will fail only whether all disjoint paths fail.

#### 3.5.3. LEACH Protocol

**Figure 15.**
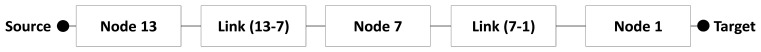
Region Model created when WSN uses the LEACH protocol.

While previous protocols consider that the WSN is flat, the LEACH protocol [26,27] periodically creates clusters by electing a leader of the cluster (CH) to receive packets from sensor nodes and forwards them to the sink node. In this way, any node (*Node 7* and *Node 13*) is one or two hops away from the sink node (*Node 1*) as shown in Figure 15.

In this case, the Region model has three node blocks as *Node 13* is not a CH (see Figure 15). The first block (*Node 13*) represents the node belonging to the region, the second block (*Node 7*) is the CH, and the last block (*Node 1*) is the sink node.

Table 1 briefly presents some differences between the three aforementioned protocols. The FLOODING protocol uses multipath to deliver a packet, which means that even if one of the paths fails, the packet is successfully transmitted. On the other hand, this protocol uses more sensor nodes and, therefore, consumes more energy.

By adopting the DIRECT protocol, packets are directly sent to the sink node, which usually decreases the WSN power consumption. However, nodes that are distant from the sink node consume more energy and usually die first. Finally, LEACH creates clusters in WSNs. This protocol helps to decrease the WSN power consumption due to the use of a cluster header (CH), whilst the CH can become a central point of failure.

### 3.6. Tooling

Due to the sequence of steps required (see Section 3.1), the Region model is difficult to create and evaluate manually. A better alternative is to use a tool that performs these tasks in an automatic way. For that reason, the proposed environment to evaluate the power consumption of the network [15,16] has been extended to also assess the reliability of the network. First, we present the proposed extensions in the environment to create and evaluate the reliability models and, next, we show how to evaluate the power consumption and reliability together.

#### 3.6.1. Proposed Extensions

**Figure 16.**
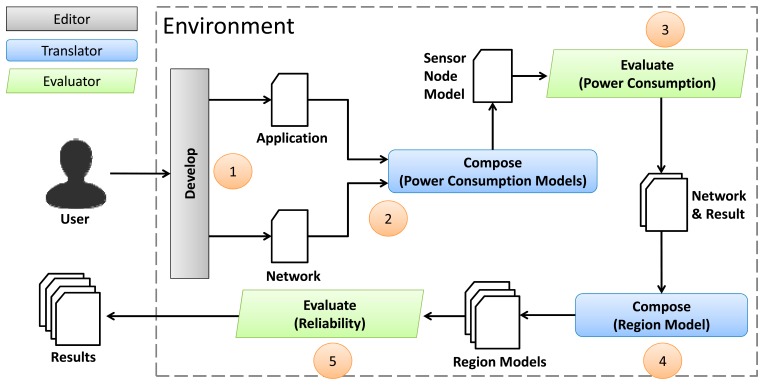
Step-by-step to evaluate the reliability of WSNs.

The tooling developed for evaluating the power consumption (see Figure 2) was extended to enable us to evaluate the reliability of WSNs. This environment is shown in Figure 16 and consists of three tools: *editor*, *translator* and *evaluator*.

The *editor* is a graphical tool that allows to implement a WSN application and create a WSN topology (e.g., by choosing the number and location of the nodes, radio range, packet size, and communication protocols). Additionally, the user defines some parameters (e.g., stop criteria, application code and WSN topology) to evaluate the power consumption of the WSN via the *editor*. However, the evaluation of the WSN reliability needs further parameters, which were added in the *editor*. The user can define a region in a WSN and set needed properties about reliability (e.g., link reliability). As before the reliability extension, the *editor* still uses the *translator* and *evaluator* to create and evaluate, respectively, the WSN power consumption and reliability.

The *translator*, as mentioned in Section 2.2, generates power consumption models of applications and networks. With the proposed extension, the *translator* also generates reliability models in RBD. The reliability of links and nodes is expressed by RBD basic blocks (see Section 3.2) automatically generated from both the WSN topology and information defined by the user in the *editor*. Meanwhile, the *translator* also generates the Path model using the defined routing protocols (see Section 3.3) and creates Region model (see Section 3.4) by composing these Paths.

Finally, while the *evaluator* assessed only the power consumption (Sensor Node, Application and Network models) using CPN Tools [17–19], with the proposed extension, it can evaluate the Region models created by the translator. Additionally, it was necessary to use another software (Mercury) [28], because the CPN Tools only deal with CPN models (and Region models are created in RBD).

#### 3.6.2. Evaluating the Reliability

**Figure 17.**
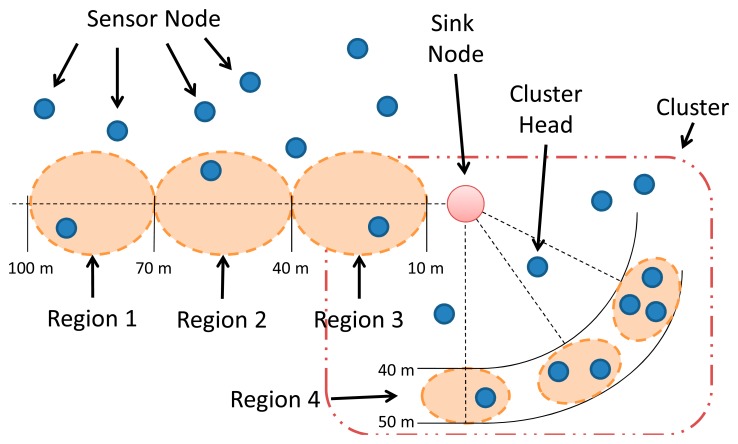
General overview of the three scenarios.

As shown in Figure 17, to evaluate the reliability of a particular WSN, an user initially needs to implement an application (in nesC) and configures the network parameters using the *editor* (Step 1): the reliability of links, applications, operating system, radio, hardware and middleware; and regions and routing protocol used. Next, the *translator* is used to create the power consumption models (Step 2) and the *evaluator* calculates the power consumption (Step 3). These two steps were showed in details in Figure 2. When the evaluation finishes, the results are stored and the network topology is used to create the region model. This topology has information provided by the user (e.g., link reliability, regions and routing protocol) and *evaluator* (e.g., battery level and if radio is on or off of all nodes), which collected these data after the evaluation of the power consumption. Next, the *translator* generates the region model from the WSN topology (Step 4). This step is divided into three sequential tasks: the *translator* converts links and nodes into basic blocks (using data defined by the user and *evaluator*), executes the routing algorithm (which was implemented inside of the *translator*) used by the WSN to create Path models, and combines them to create the Region models. In addition, for example, if a topology has 10 regions, the *translator* creates 10 Region models. It is necessary to evaluate each region individually (as mentioned in Section 3.4). Finally, in Step 5, the Region Models are evaluated (by generating reliability results to each Region model created) and the results are presented (reliability of the regions and power consumption of the application and network) to the user.

Finally, this environment is a Web application developed in Java, has been deployed at Amazon EC2 [29] and its source code is available in the GitHub [30].

## 4. Experimental Evaluation

In order to evaluate the proposed models, we carried out an experimental evaluation in three different scenarios. First scenario (*Scenario 1*) evaluates the reliability of a single region using different routing protocols (FLOODING, DIRECT and LEACH). Second scenario (*Scenario 2*) assesses the reliability of three different regions (1, 3 and 5 nodes) using the same protocol (LEACH). Finally, the last scenario (*Scenario 03*), evaluates the reliability of three different regions that use the same routing protocol (DIRECT), but whose distances to the sink node is variable. Using these scenarios, it is possible to observe that the reliability of a particular region is affected by the routing protocol (*Scenario 1*), by the number of nodes belonging to the region (*Scenario 2*) and by the distance from these regions to the sink node (*Scenario 3*).

These scenarios were evaluated using a WSN including 20 sensor nodes (with energy equal to 1 mJ) and one sink node. The distance between the sink node and other nodes ranges from 10 to 50 m (*Scenario 1* and *Scenario 2*) and from 10 to 100 m (*Scenario 3*). This setup was also used by Senouci *et al.* [24], who evaluated the power consumption of different protocols. However, it is not interesting to assess the effect of distance on the WSN reliability because the sensor nodes were very close. For that reason, it was necessary to change the distance between the sensor nodes and the sink node in the *Scenario 3*.

**Figure 18.**
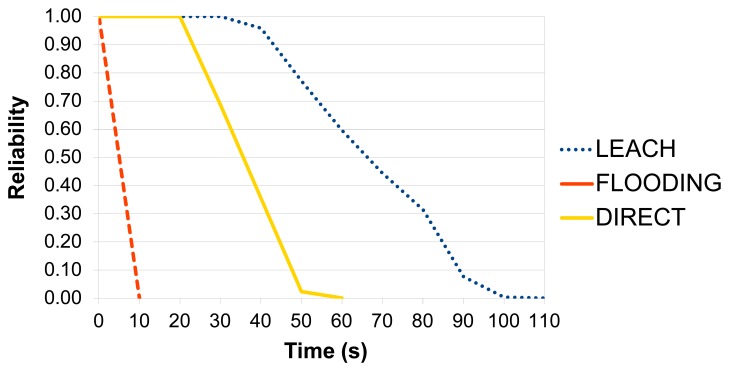
Reliability of a region using three different routing protocols.

As shown Figure 18, four regions were created in different positions of the WSN: Regions 1, 2 and 3 were used in *Scenario 3*; and Region 4 was used in *Scenarios 1* and *Scenario 2*. The reliability of the Basic Block elements (Link, App, OS, Middleware, Hardware and Radio) was set to “1”, whilst the reliability of the battery was set after evaluating the power consumption models as presented in the next subsection.

### 4.1. Battery Reliability

The battery reliability has been defined through an experiment using actual motes (nodes). The relationship between the reliability and the battery level is determined by averaging errors occurring in a given voltage. To adjust the voltage, a source of DC voltage was used. It is worth observing that the decrease of the battery over time was not considered in this experiment, because voltage does not decrease along the time like battery. It is necessary to change the voltage manually using the source of DC voltage. Additionally, this experiment used the IRIS mote, which works properly between 3.3 V and 2.7 V (MEMSIC, 2014) [31], *i.e.*, it is able to send a packet without failures.

This experiment used a sensor node to periodically send messages to a desktop that detects whether an error occurred in the sensor node or not. An application was developed and deployed in the mote to collect the temperature and send it to the sink node, which forwards it to the desktop. The communication between the sensor node and the sink node is direct and the distance is short enough to avoid interference. Any error in the packet is only due to the sensor node.

The aforementioned application was evaluated with different battery levels, starting from 3.3 V (battery level equal to 100%) to 2.7 V (representing 81.81% of the battery level). It is worth noting that these levels are informed by the IRIS manufacturer for the proper functioning of the mote. Additionally, the voltage was also adjusted from 2.7 V until the sensor node stops working. It was observed that the sensor node also works correctly from 2.7 V (81.82% of battery level) to 1.78 V (53.94% of battery level), *i.e.*, the sensor node did not fail in this voltage interval.

With these initial results, we created a strategy to define battery reliability using proportion, because it is interesting to work with the possibility of failure due to the battery level. For that reason, the reliability of the battery is 1.0 when the battery level ranges from 100% (3.3 V) to 81.81% (2.7 V) and set to 0.0 when the battery level is below 53.94% (1.78 V). When the battery level ranges from 81.81% to 53.94%, we will use a proportion of the battery reliability $\left(\frac{R}{1}=\frac{\text{Level}-\mathrm{Min}}{\mathrm{Max}-\mathrm{Min}}\right)$.

Thus, the battery reliability is configured according to Equation (9):

(9) $$R_{\text{battery}}(\text{Level})=\{\begin{array}{ll} 1 & \text{if level ranges from}100\%to81.81\%\\ \frac{\text{Level}-\mathrm{Min}}{\mathrm{Max}-\mathrm{Min}} & \text{if level ranges from}81.81\%to53.94\%\\ 0 & \text{if Level is below}53.94\% \end{array}$$

where *Max* and *Min* are equal to 0.8182 (81.82% of battery level) and 0.5394 (53.94%), respectively; and *Level* is a value of the battery level. For example, if the battery level is equal to 70% (0.70), its reliability is 0.57. It is worth observing that, as shown in this equation, the reliability of the battery itself is not influenced by any other component of the sensor node, e.g., radio, hardware, application, middleware and operating system.

### 4.2. Obtained Results

As mentioned before, *Scenario 1* shows the impact of the routing protocol on the region reliability, *i.e.*, a single region and three different routing protocols: FLOODING, DIRECT and LEACH. The WSN reliability of each protocol is illustrated in Figure 18. Additionally, the strategy defined in Section 4.1 was considered.

As expected, the WSN reliability is low when the FLOODING protocol is used as its power consumption is very high. The WSN reliability using DIRECT is lower than that of LEACH because the distance from the region to sink node has great impact on DIRECT than other protocols: the furthest nodes die sooner when the network uses the DIRECT protocol. Meanwhile, LEACH uses clusters that help to decrease the power consumption, balancing the power consumption of the network. Even though LEACH involves more links and sensor nodes in the path (which can decrease the reliability), the battery level is the key factor being considered. Additional factors, such as the quantity of sensor nodes and setup time, could be also considered.

It is worth observing that FLOODING becomes a good alternative in the presence of failures of the sensor nodes and communication links, and when the sensor has enough energy. To show this situation, we carried out an experiment using *Scenario* 1, but considering the following setup: the battery level is constant (battery reliability set to 1.0); and the reliability of the sensor nodes and links was set to 0.1, *i.e.*, they have a very low reliability.

**Figure 19.**
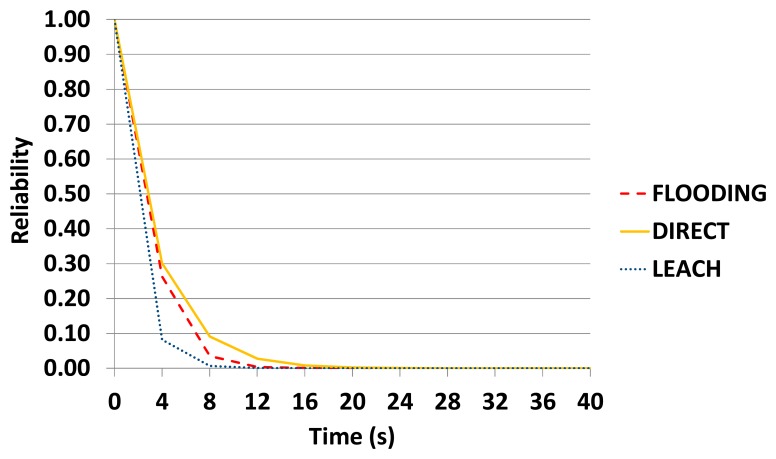
WSN reliability when the sensor has enough energy.

Figure 19 shows the results of this experiment. The DIRECT protocol had the best result, because it involved only two sensor nodes and one link. Furthermore, the reliability of the link does not consider the distance between the transmitter and receiver, what helped the DIRECT results. FLOODING had the second best result, because it created multiple paths, and LEACH had the worst result, because it used more sensor nodes than DIRECT and had a bottleneck (Cluster Head).

*Scenario 2* evaluates the impact of the number of sensor nodes of a region on its reliability. In this case, we consider a region containing 1, 3 and 5 nodes, and we adopted the LEACH routing protocol because it creates clusters in the network, which is ideal to evaluate the cooperation among sensor nodes. Finally, we also used the strategy defined in Section 4.1.

**Figure 20.**
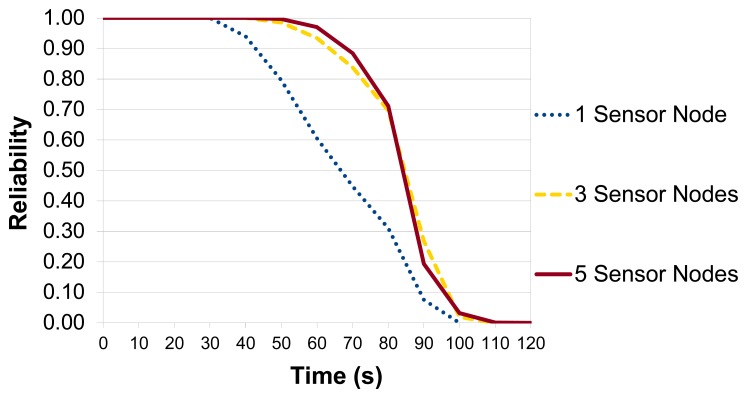
Reliability of the region considering different number of nodes.

As illustrated in Figure 20, the region with 1 sensor node has the worst reliability, whilst the difference between regions with 3 and 5 sensor nodes was not significant because they have a bottleneck in common (CH). If the CH fails, the region fails no matter the number of sensor nodes inside because the paths have the same CH as a common point (see Figure 15). However, the region with *5* sensor nodes was available for a longer time, because its reliability is only equal to zero when the battery level of all sensor nodes is less than 53.94%.

Last scenario, *Scenario 3*, shows that the location of the region impacts on its reliability. For that reason, the sensor nodes were placed between 10 to 100 m from the sink node. Each region has a single node and the DIRECT protocol was adopted. We choose this protocol, because the farthest sensor nodes die earlier when using this protocol (as mentioned before).

**Figure 21.**
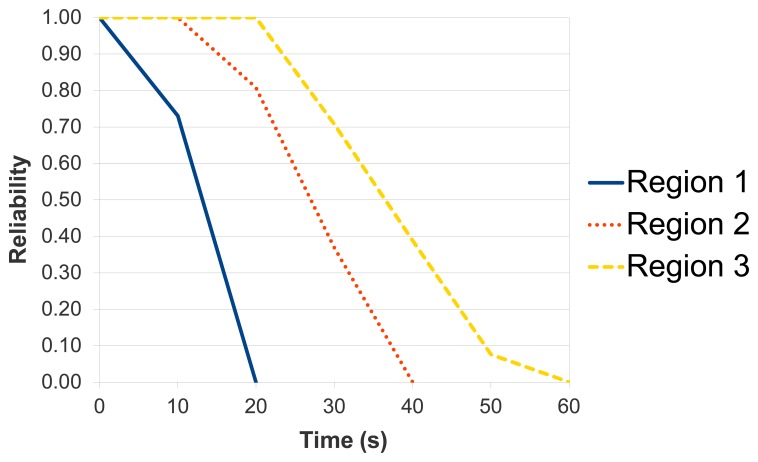
Reliability of the region in three different positions.

As shown in Figure 21 (considering the strategy defined in Section 4.1), the reliability of the region closer to the sink node is the highest, whilst the reliability of the further region is the worst one. Moreover, this scenario highlights the importance of evaluating individual regions of a WSN instead of the entire network.

## 5. Related Work

Related works have been organized according to three different evaluation techniques: measurement, simulation and analytical modeling. The measurement method analyzes the reliability directly using an actual sensor node (mote). Researches on measurement usually determine the reliability of a WSN based on the rate of number of sent messages and number of received messages in sink node. For example, Zhao and Govindan [4] evaluated the impact of the physical and MAC layers on the reliability of a WSN. Additionally, they evaluated the WSN reliability in different conditions (e.g., indoor and outdoor). In another example, Korkmaz and Sarac [32] used measurement to discover the link reliability. Next, they apply these results to evaluate the reliability of a WSN with re-transmissions and multipath routing.

Existing works on assessing the WSN reliability through simulation use models for simulating propagation and interferences in the signal. There are propagation models that do not consider packet lost caused by the environment [33] and specific models for WSNs that consider irregular propagation and interference by other elements (e.g., Radio Irregularity Model [5]). These models are added to the simulators to allow to better evaluate situations of packet lost, collision or errors in the WSN. For instance, Wang *et al.* [6] proposed a WSN congestion control protocol called Priority-based Congestion Control Protocol (PCCP). Through simulation, it was possible to evaluate the benefits of avoiding and decreasing the packet lost in the WSNs, increasing the reliability of the sensor nodes.

The last technique (analytical modeling) assesses the reliability or availability of WSNs when we know the fault rate and the probabilities of their occurring. We can use three different ways to evaluate the reliability using analytical modeling: Fault Tree, Markov Chain and RBD. Fault Tree is a graphical model (using logic gates) that represents events (with conditions to happen) and their consequences, which lead to the system failure [9]. Markov Chain considers that the system has states (e.g., working state and failure state) and its state varies with the time. It can be represented by a directed graph, where the vertices are states and the edges show the possibilities of change between states. Additionally, the edges have a probability (assigned on the label) of transition from one state to another.

Silva *et al.* [9] proposed a methodology for evaluating the reliability and availability of WSNs using Fault Tree. The proposed methodology evaluates different kinds of networks (e.g., single hop or multiple hop). However, this approach does not consider the routing protocols. Instead of this, the network is converted into a graph and the depth-first search is used to find a path between the sensor nodes. The model proposed in this paper is close to one introduced by Silva [9]. However, this proposal considers that the routing protocol is essential to create the paths between the sensor nodes and sink node instead of using depth-first search. Besides that, our proposal also introduces the notion of region, which can be evaluated individually. Finally, the battery level is considered the main reason to sensor node faults and its value is obtained by evaluating the power consumption.

Bein *et al.* [7] evaluated the WSN reliability using Markov Chain and RBD and show how to increase the network reliability by replacing the faulty sensor nodes. However, WSNs usually do not allow to repair or replace the sensor nodes due to the environment hostility (e.g., close to volcanoes) or difficulty to their access (e.g., dense forests).

Ghaffari *et al.* [8] used RBD for evaluating the reliability of two transport protocols, namely Event-to-sink Reliable Transport (ESRT) [34] and Reliable Multi-Segment Transport (RMST) [35], where the packets are sent in broadcast and paths have the same reliability level. Protocol RMST has reliability better than ESRT, because it retransmits a packet many times. However, retransmitting a packet increases the power consumption (by decreasing WSN lifetime) and increases the traffic (by increasing the chance of the collision among the packets). These two features were not considered in this study. Additionally, we work with protocols of network layer, whilst Ghaffari concentrates on transport layer protocols.

Zonouz *et al.* [36] evaluated the WSN path reliability using only Equation (4). They evaluated the reliability and power consumption of WSNs. Like our approach, they consider that WSNs have two main points of failure: links and sensor nodes. The probability of a link failure is determined by the distance between the transmitter and the receiver, while we associated a value independent from the distance between them. In terms of the node failure, each node has a constant failure rate of 5.0^−5^ failures per hour, while we explicitly consider the sensor node failure associated to a series of elements (e.g., battery, application, middleware, operating system and so on). In practice, they do not consider the impact of the battery level on the sensor node reliability. Finally, they also consider routing protocols (two single-paths) in their approach, whilst we evaluate three protocols (multipath and two single-paths).

Finally, Wang *et al.* [37] proposed an analytical and combinatorial method to evaluate the reliability of multi-state systems (like WSNs). However, despite its potential, it has not been applied to evaluate the reliability of WSNs.

## 6. Conclusion and Future Work

This paper presented a WSN reliability model that is generated automatically from the WSN topology and information about adopted routing algorithms and the mote battery level. This model considers that WSN can fail in two points: links and sensor nodes. The proposed models were evaluated in three scenarios. Using these scenarios, it was possible to observe that the reliability of a particular region is affected by the routing protocol adopted, by the number of nodes belonging to the region and by the distance of these regions to the sink node.

This paper has three main contributions related to the evaluation of WSN: it considers the mote energy level as the main factor of failures of WSN nodes; it uses the routing algorithm to define the paths between different WSN regions and the sink node; and it automatically generates reliability models considering the aforementioned elements.

As future work, two main steps are now starting to be developed: to consider the reliability of additional communication protocols (e.g., B-MAC and RSMT); and to extend the current tooling in such way that it becomes able to suggest improvements in the WSN in order to increase the WSN reliability.

## Figures and Tables

**Table 1.** Comparative elements of routing protocols.

**Protocol**	**Communication**	**Multipath**	**Number of Hops to the Sink Node**
FLOODING	broadcast	YES	Undefined
DIRECT	unicast	NO	1
LEACH	unicast	NO	1 or 2
